# Effects of Surfactant Volume Fraction on the Antioxidant Efficiency and on The Interfacial Concentrations of Octyl and Tetradecyl *p*-Coumarates in Corn Oil-in-Water Emulsions

**DOI:** 10.3390/molecules26196058

**Published:** 2021-10-07

**Authors:** Marlene Costa, Sonia Losada-Barreiro, Fátima Paiva-Martins, Carlos Bravo-Díaz

**Affiliations:** 1 REQUIMTE/LAQV, Department of Chemistry and Biochemistry, Faculty of Science, University of Porto, Rua do Campo Alegre 687, 4169-007 Porto, Portugal; marlene.andreia.costa@gmail.com (M.C.); mpmartin@fc.up.pt (F.P.-M.); 2 Departamento de Química-Física, Facultad de Química, Universidade de Vigo, 36310 Vigo, Spain; sonia@uvigo.es

**Keywords:** cinnamic acid, emulsions, oxidative stability, interfacial concentrations

## Abstract

Surfactants have been used for decades in the food industry for the preparation of lipid-based emulsified food stuffs. They play two main roles in the emulsification processes: first they decrease the interfacial tension between the oil and water, facilitating droplet deformation and rupture; second, they reduce droplet coalescence by forming steric barriers. However, addition of surfactants to binary oil-water mixtures also brings up the formation of three-dimensional interfacial layers, surrounding each emulsion droplet, that significantly alter chemical reactivity. This is the case, for instance, in the inhibition reaction between antioxidants and the lipid radicals formed in the course of the spontaneous oxidation reaction of unsaturated lipids, which are commonly employed in the preparation of food-grade emulsions. The rate of the inhibition reaction depends on the effective concentrations of antioxidants, which are mostly controlled by the amount of surfactant employed in the preparation of the emulsion. In this work, we analyze the effects of the surfactant Tween 20 on the oxidative stability and on the effective concentrations of two model antioxidants derived from cinnamic acid, determining their interfacial concentrations in the intact emulsions to avoid disrupting the existing equilibria and biasing results. For this purpose, a recently developed methodology was employed, and experimental results were interpreted on the grounds of a pseudophase kinetic model.

## 1. Introduction

**Scheme 1:**
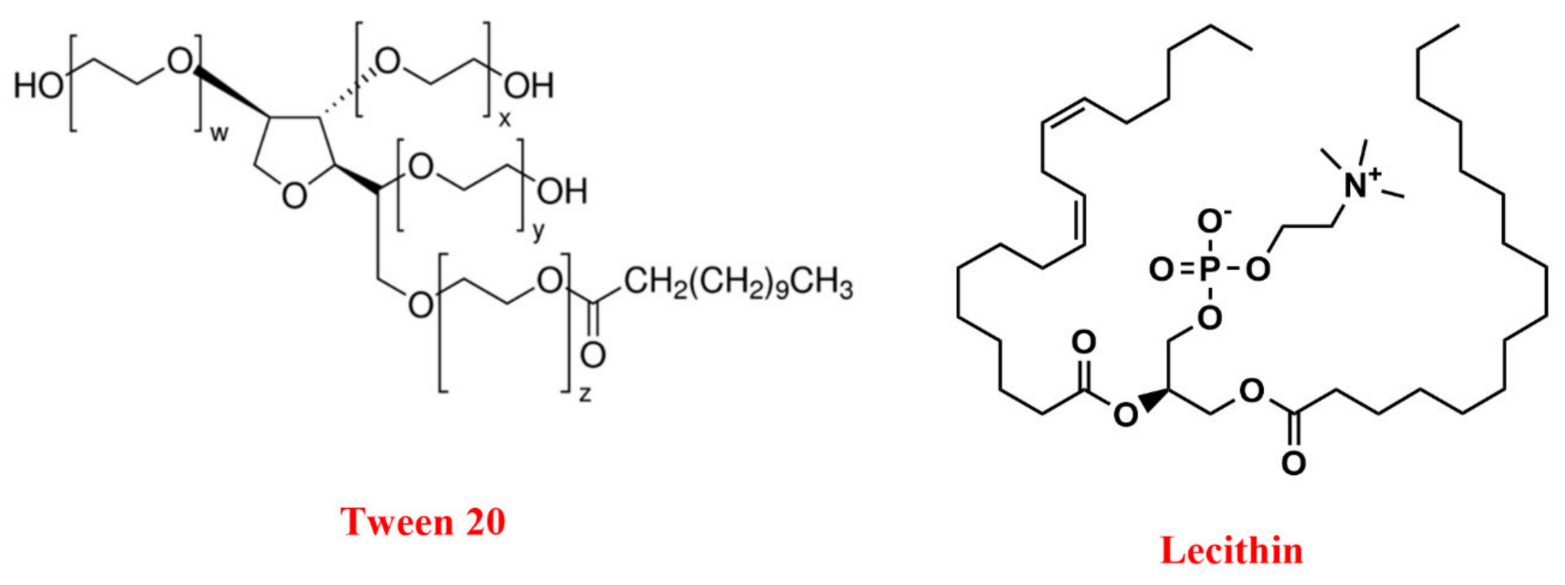
Chemical structures of nonionic and zwitterionic surfactants commonly employed in the preparation of foodstuffs. Anionic surfactants such as sodium dodecyl sulphate (SDS) are less frequently used to avoid electrostatic repulsions with the electrolytes present in water (e.g., NaCl, H_3_O^+^), and cationic surfactants such as cetyltrimethylammoium bromide (CTAB) are never used.

Naturally occurring surfactants, such as proteins from milk and egg lecithins, have been employed for years in the preparation of lipid-based foods such as mayonnaise, salad creams, dressings, etc. [1,2,3]. More recently, small synthetic surfactants such as sorbitan esters, their ethoxylated derivatives, and sucrose esters have been incorporated in the formulation of lipid-based food emulsions [4,5]. Scheme 1 shows some common surfactants employed in the food industry.

Emulsions are widely used in the preparation of emulsified foods because lipids are mostly present in foods in the form of oil-in-water emulsions, where the oil droplets are dispersed in an aqueous solution. Other emulsions that are employed include water-in-oil emulsions (w/o), where water droplets are dispersed in an oil, and water-in-oil-in-water (w/o/w) emulsions, which consist of an o/w emulsion whose droplets themselves contain water droplets. The o/w emulsions are probably the most versatile, and their properties are controlled by both the surfactants and the composition of the aqueous phase. They are present in many dairy foods, including mayonnaise, creamers, whippable toppings, ice creams, etc. [4]. Butter, margarines, and fat-based spreads are typical examples of w/o emulsions, and their properties depend largely on the properties and particular composition of the fats or oils and the surfactant used in the water phase. Table 1 shows examples of typical food emulsions [1,6].

Emulsions are thermodynamically unstable systems, but they can be stabilized kinetically for some time (minutes to years) by adding surfactants that are adsorbed at the oil-water interface, creating a narrow (2–20 nm thick) three-dimensional layer surrounding the emulsion droplets and causing repulsive forces that provide some kinetic stability. Neutral surfactants mainly create steric barriers that prevent emulsion droplets from breaking down. Meanwhile, the ionic ones act through electrostatic repulsions, preventing droplets from approaching each other [7]. The emulsifying capability of an agent can be assessed through the hydrophile-lipophile balance (HLB), which is defined as the ratio of the weight percentage of hydrophilic groups to the weight percentage of hydrophobic groups in the molecule. HLB values for commercial emulsifying agents range from 1 to 20. Surfactants with low values, 3–6, promote the formation of water-in-oil emulsions (glycerol esters, propylene glycol fatty acid esters, polyglycerol esters, and sorbitol fatty acid esters). Meanwhile, those with HLB values > 10 promote the formation of oil-in-water emulsions. Table 2 shows the HLB of some common surfactants and their main uses [1,4,7].

**Scheme 2:**
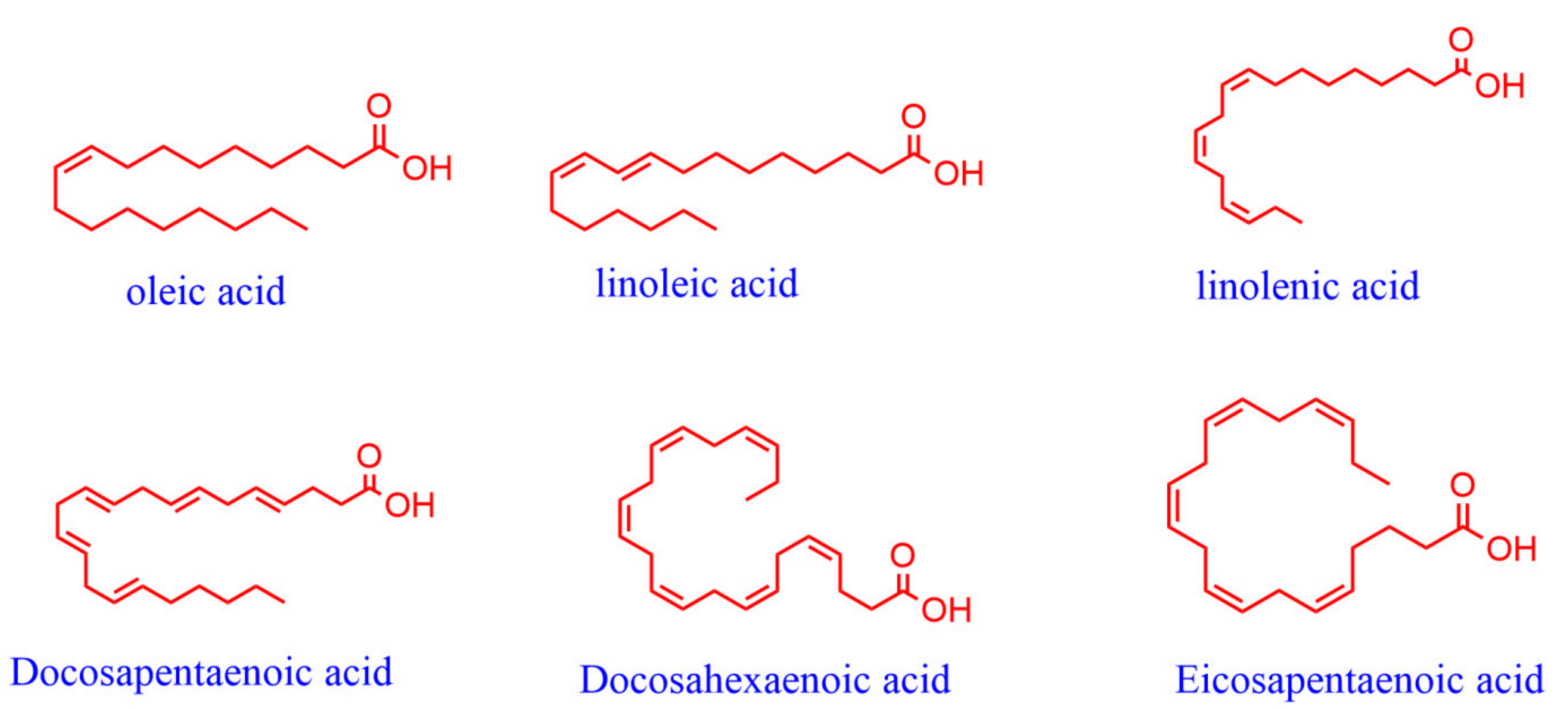
Some common unsaturated fatty acids present in vegetal (olive, corn, etc.) and animal (fish) oils. Oleic acid is the main fatty acid present in olive oil; meanwhile eicosapentaenoic acid is present in omega-3 rich fish oils.

The oils commonly employed in the preparation of food emulsions contain unsaturated lipids, which are prone to oxidation, leading to the development of off-odors, the production of potentially harmful products and, in general, to a loss of the nutritional and organoleptic properties of the food [9,10,11]. Scheme 2 shows the chemical structures of some of the most common unsaturated fatty acids found in food-grade oils. 

Control of the lipid oxidation reaction is, therefore, a challenge not only for the food industry but also for the pharmaceutical industry, where unsaturated lipids are employed in the formulation of products of interest, such as the nutritive emulsions employed in parenteral nutrition, and for the cosmetics industry, where they are widely used in the preparation of lotions, beauty creams, etc. One of the most effective ways of inhibiting the oxidation of lipid-based foods is the incorporation of antioxidants, AOs, which are molecules capable of donating H-atoms to lipid radicals to regenerate them [4,12,13]. The efficiency of the AOs in inhibiting lipid oxidation is usually assessed in terms of the rate of the chemical reaction between the AO and the lipid radical, which depends on, among others, the effective concentrations of the antioxidant in the interfacial region of the emulsions [13,14,15,16,17,18].

**Scheme 3:**
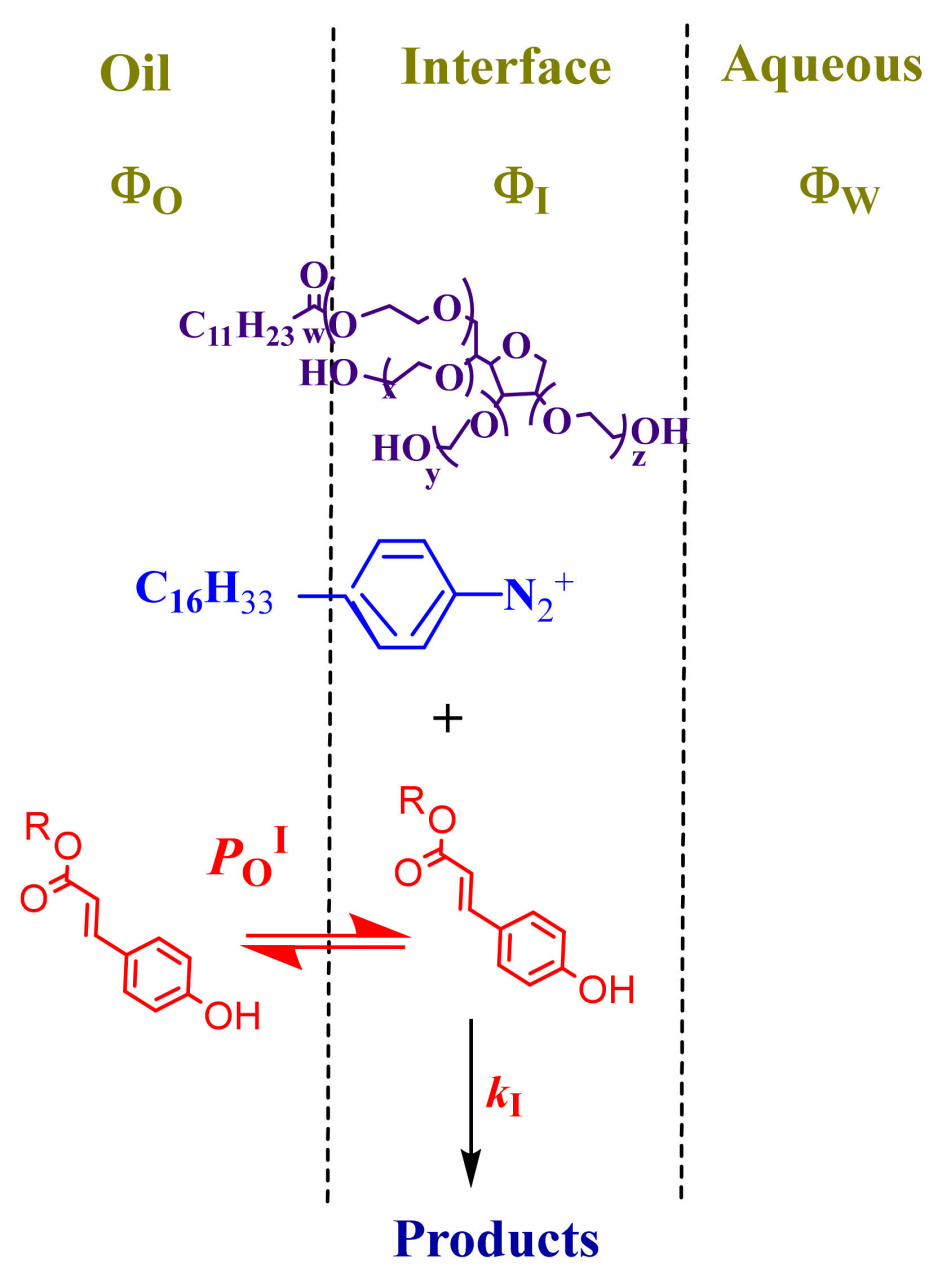
Representation of the oil (O), water (W), and interfacial (I) regions of emulsion droplets, showing the emulsifier Tween 20 and the location of the reactive probe 16-ArN_2_^+^. Antioxidants of moderate hydrophobicity distribute between the three regions, but for the sake of simplicity and clarity, only the distribution of hydrophobic AOs, such as those employed in this work (see experimental section), is shown. Φ is the volume fraction of a region, *P*_O_^I^ is the partition constant between oil and interfacial regions, and *k*_I_ is the rate constant for reaction between 16-ArN_2_^+^ and the antioxidants in the interfacial region. See text for further details.

However, determining concentrations of AOs at the interfaces of emulsions is challenging because of the physical impossibility of separating the interfacial region from the oil and aqueous ones without disrupting the existing equilibria [19,20]. Our laboratory developed a kinetic method to determine the effective concentrations of antioxidants in emulsions. The method is based on the reaction of the chemical probe 4-hexadecylbenzenediazonium ion, whose reactive group (-N_2_^+^) is exclusively located in the interfacial region of emulsions with the antioxidants; see Scheme 3. The experimental results were then interpreted on the grounds of the pseudophase kinetic model [19]. The basis of the method has been published, and only a brief explanation will be given here (Section 1.1). 

Here, we determined the distribution and efficiencies of *p*-octyl- and *p*-tetradecylcoumarates in corn oil-in-water emulsions stabilized with Tween 20. Their chemical structures are given in Scheme 3. We also determined their antioxidant activity in the same intact emulsions by monitoring the formation of conjugated dienes with time. Coumaric acid derivatives are of interest for humans because they can decrease low density lipoprotein (LDL) peroxidation and reduce the risk of stomach cancer showing anti-mutagenesis, anti-genotoxicity, and anti-microbial activities, inhibiting cellular melanogenesis [21,22,23]. However, their efficiency is, in general, low because their solubility in the interfacial and oil regions of lipid-based systems is quite low; however, in some instances, they can be improved by esterification of their carboxylic group, increasing their hydrophobicity [24,25,26,27].

### 1.1. Determining Antioxidant Distributions in the Intact Emulsions: Application of the Pseudophase Kinetic Model

Because it is physically impossible to separate the interfacial region from the oil and aqueous ones without disrupting the existing equilibria, the distribution of AOs needs to be assessed in the intact emulsions to avoid disruption of the existing equilibria [13,19,20]. The method developed focuses on determining the partition constants of antioxidants between the aqueous-interfacial, *P*_W_^I^, and oil-interfacial, *P*_O_^I^, regions of the emulsions. For this purpose, a kinetic method that exploits the chemical reaction between a hydrophobic arenediazonium ion, 4-hexadecylbenzenediazonium, 16-ArN_2_^+^, and the antioxidants was developed; see Scheme 3 [20,27,28,29,30,31,32]. The chemical probe 16-ArN_2_^+^ is itself a water and oil insoluble ionic surfactant, with the reactive –N_2_^+^ group located exclusively in the interfacial region of the emulsions, where it reacts with the antioxidant as illustrated in Scheme 3. Thus, its effective concentration in the oil and water regions is null, and the rate of the reaction of 16-ArN_2_^+^ with antioxidants will depend only on the measured (or observed) rate constant, *k*_obs_, and on the concentrations of 16-ArN_2_^+^ and antioxidant, AO, in the interfacial region, as in Equation (1):

*v* = *k*_obs_[1 6-ArN_2_^+^_T_] = *k*_2_[16-ArN_2_^+^_T_][AO_I_] = *k*_I_(16-ArN_2_^+^_T_)(AO_I_)Φ_I_ (1)

where *k*_2_ and *k*_I_ are, respectively, the observed second order rate constant and the second order rate constant in the interfacial region; square brackets, [ ], denote the concentration in mol per liter of total emulsion volume; parentheses, ( ), indicate concentration in mol per liter of the volume of a particular region; subscript T stands for the stoichiometric or total concentration; subscripts O, I, and W indicate the oil, interfacial, and aqueous regions, respectively; and Φ_I_ = V_surf_/V_emulsion_ is the surfactant volume fraction, defined as Φ_I_ = V_surf_/V_Total_, which is assumed to be equal to that of the interfacial region.

The distribution of the hydrophobic AOs employed in this work (Scheme 3) is defined by the partition constant between the oil-interfacial, *P*_O_^I^ (Equation (2)) [33,34,35], and the observed rate constant is given by Equation (3). Experiments are usually carried out at constant [AO_T_] and Φ_O_ = V_oil_/V_emulsion_, so that the *P*_O_^I^ values can be obtained from the experimental variation of 1/*k*_obs_ *vs* Φ_I_, Equation (4) (which is the reciprocal form of Equation (3)), and that predicts that plots should be linear with positive intercepts, from which the partition constant values can be obtained.

(2) $$P_{O}^{I}=\frac{{(\mathrm{AO}}_{I})}{{(\mathrm{AO}}_{O})}$$

(3) $$k_{\mathrm{obs}}=\frac{k_{I}{\left[\mathrm{AO}\right]}_{T}P_{O}^{I}}{\Phi_{I}P_{O}^{I}{+\Phi }_{O}}$$

(4) $$\frac{1}{k_{\mathrm{obs}}}=\frac{\Phi_{O}}{k_{I}{\left[\mathrm{AO}\right]}_{T}P_{O}^{I}}+\frac{1}{k_{I}{\left[\mathrm{AO}\right]}_{T}}\Phi_{I}$$

Once the *P*_O_^I^ values are known; the percentages and effective concentrations of the AOs in the interfacial and oil regions can be determined by employing Equations (5)–(8). Details on these calculations, as well as the equations for antioxidants of moderate and low hydrophobicity, can be found elsewhere [33,34,35,36,37,38].

(5) $${\%\mathrm{AO}}_{I}=\frac{100P_{O}^{I}\Phi_{I}}{\Phi_{I}P_{O}^{I}{+\Phi }_{O}}$$

(6) $${\%\mathrm{AO}}_{O}=\frac{{100\Phi }_{O}}{\Phi_{I}P_{O}^{I}{+\Phi }_{O}}$$

(7) $${(\mathrm{AO}}_{I})=\frac{{[\mathrm{AO}}_{I}{](\%\mathrm{AO}}_{I})}{\Phi_{I}}$$

(8) $${(\mathrm{AO}}_{O})=\frac{{[\mathrm{AO}}_{I}{](\%\mathrm{AO}}_{O})}{\Phi_{O}}$$

## 2. Experimental Materials and Methods

### 2.1. Materials

All chemicals were of the highest purity available and were used as received. Meldrum’s acid, benzaldehyde, 1-octanol, 1-tetradecanol, β-alanine, and pyridine were purchased from Sigma-Aldrich (Darmstadt, Alemania, and polyoxyethylene (20) sorbitan monolaurate (Tween 20) from Fluka (Buchs, Switzerland). Stripped corn oil (Acros Organics, Geel, Belgium) was used as received and kept at low temperature in the dark to minimize lipid peroxidation. Distilled and de-ionized water (conductivity < 0.1 μS cm^−1^) was used in all experiments. The acidity of aqueous phase was controlled by employing citric acid/citrate buffer (0.04 M, pH 3.65). Solutions of the coupling agent *N*-(1-Naphthyl)ethylenediamine (NED, Aldrich, Darmstadt, Alemania) were prepared in a 50:50 (*v*/*v*) BuOH:EtOH mixture to give [NED] = 0.02 M. The 4-hexadecylbenzene diazonium tetrafluoroborate, 16-ArN_2_BF_4_, was prepared under nonaqueous conditions as described in a published method [39] from commercial 4-hexadecylaniline (Aldrich, 97%) and was stored in the dark at low temperature to minimize decomposition.

All reactions were monitored by thin layer chromatography (TLC) on precoated aluminum silica gel sheets 60 F254 plates (Merck, Darmstadt, Germany), and spots were detected using a UV lamp at 254 nm and iodine. Products were purified by chromatography with silica gel 60 (0.040–0.063 mm, Merck). Nuclear magnetic resonance (NMR) spectra were recorded on 400 or 100 NMR equipment for solutions in CDCl_3_.

### 2.2. Synthesis of Coumaric Fatty Acid Esters

**Scheme 4:**
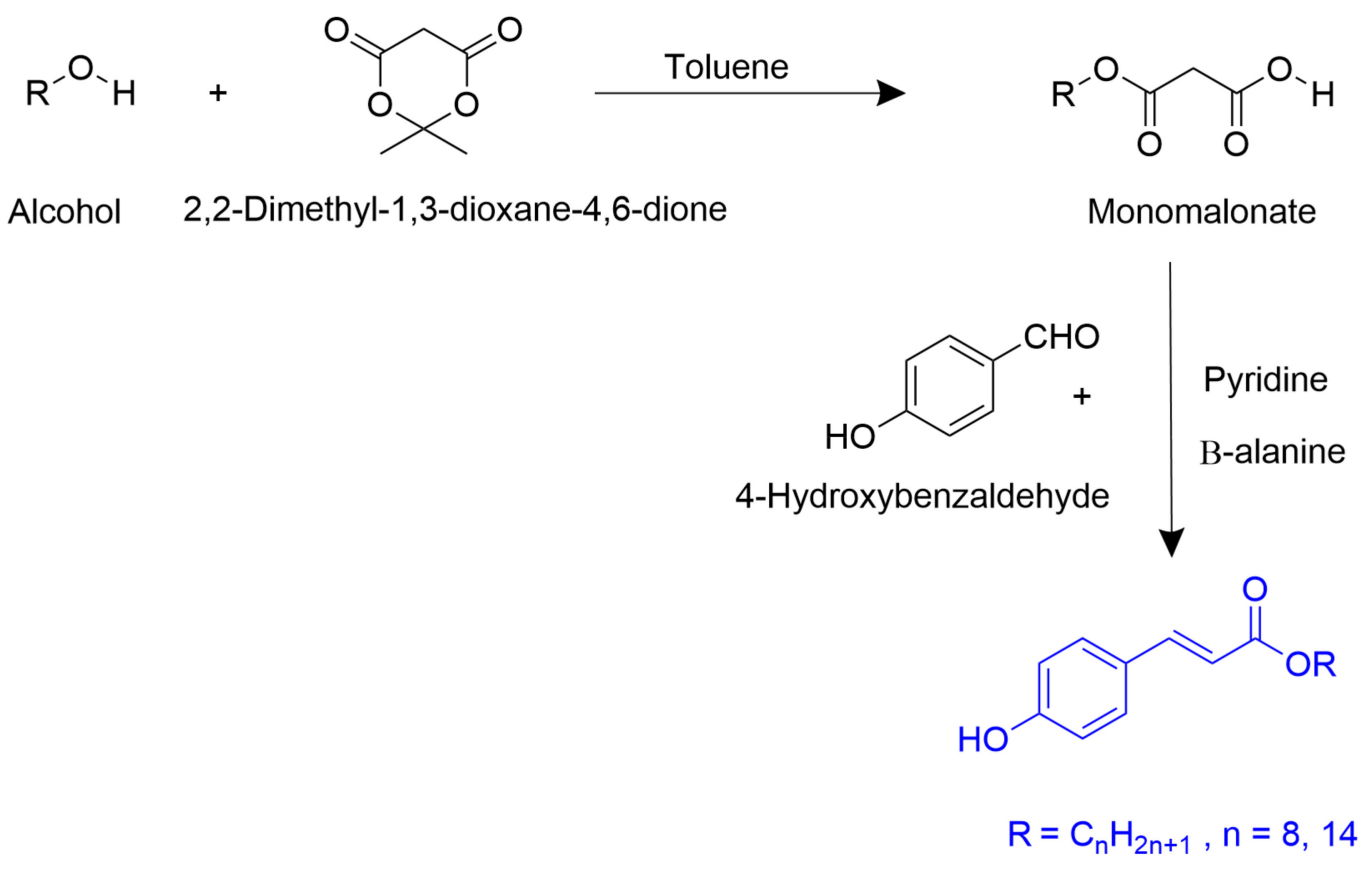
Synthesis of octyl and tetradecyl coumarates, OC and TC, respectively.

Coumaric fatty acid esters, Octyl trans-3-(4-hydroxyphenyl) propanoate (octyl coumarate, OC) and Tetradecyl trans-3-(4-hydroxyphenyl) propenoate (tetradecyl coumarate, TC) coumarates were prepared by the Verley-Doebner modification of Knoevenagel condensation, as shown in Scheme 4. Previously, appropriate monomalonates were synthesized by reaction of Meldrum’s acid with capryl and myristyl alcohol following a published procedure [40].

Briefly, the appropriate ester of malonic acid (2 mmol) was mixed with 4-hydroxybenzaldehyde (2 mmol), dry pyridine (1.0 mL), and β-alanine (15 mg) using a round-bottom flask, and the mixture was stirred for 24 h at T = 60 °C. After cooling the reaction mixture in an ice bath, HCl 37% (1 mL) was added, and the mixture was extracted with diethyl ether. Crude octyl and tetradecyl coumarates were further purified by flash column chromatography over silica gel using ethyl acetate/petroleum ether (1:1, *v*/*v*) as the eluent. The identity and purity of the malonates intermediates and the coumarates was confirmed by ^1^H and ^13^C NMR.

Octyl Malonate. ^1^H NMR (CDCl_3_, 400 MHz): δ 0.88 (t, *J* = 7.0 Hz, 3H, H-8′); 1.27 (bs, 10H, H-7′-3′); 1.65 (m, 2H, H-2′); 3.44 (s, 2H, H-2), 4.15 (t, *J* = 6.8 Hz, 2H, H-1′).

Tetradecyl malonate. ^1^H NMR (CDCl_3_, 400 MHz): δ 0.88 (t, *J* = 6.8 Hz, 3H, H-14′); 1.26 (bs, 22H, H-13′-3′); 1.66 (m, 2H, H-2′); 3.43 (s, 2H, H-2), 4.18 (t, *J* = 6.8 Hz, 2H, H-1′).

Octyl Coumarate (Octyl trans-3- (4-hydroxyphenyl) propanoate). ^1^H NMR (CDCl_3_, 400 MHz):δ 0.87 (t, *J* = 6.8 Hz, 3H, H-8′); 1,27 (bs, 10H, H-7′-3′); 1.68 (m, 2H, H-2′); 4.18 (t, *J* = 6.6 Hz, 2H, H-1′), 6.29 (d, *J* = 16.0 Hz, 1H, H-2), 6.86 (d, *J* = 8.8 Hz, 1H- H-6, 8), 7.41 (d, *J* = 8.4 Hz, 2H, H-5, 9);7.62 (d, *J* = 16 Hz, 1H, H-3).

^13^C NMR (CDCl_3_; 100 MHz): δ 13.8 (C-8′); 22.4 (C-7′); 25.8 (C-3′); 28.5 (C-2′); 29.0 (C-4′,5′); 31.6 (C-6′); 64.6 (C-1′); 115.7 (C-6,8); 126.7 (C-4); 129.8 (C-5, 9); 144.5 (C-3); 157.9 (C-7); 167.8 (C-1).

Tetradecyl Coumarate (Tetradecyl trans-3- (4-hydroxyphenyl) propenoate). ^1^H NMR (CDCl_3_, 400 MHz):δ 0.88 (t, *J* = 6.8 Hz, 3H, H-14′); 1.27 (bs, 22H, H-13′-3′); 1.71 (m, 2H, H-2′); 4.20 (t, *J*= 6.6 Hz, 2H, H-1′), 6.31 (d, *J* = 15.6 Hz, 1H, H-2), 6.86 (d, *J* = 8.4 Hz, 1H- H-6, 8), 7.44 (d, *J* = 8,4 Hz, 2H, H-5, 9);7.64 (d, *J* = 16 Hz, 1H, H-3).

^13^C NMR (CD-Cl_3_; 100 MHz):δ 14.1 (C-14′); 22.7 (C-13′); 25.9 (C-3′); 28.-7 (C-2′); 29.2 (C-4′,11′); 29.6 (C-5′-10′), 31.9 (C-12′); 64.8 (C-1′); 115.9 (C-6,8); 127.2 (C-4); 129.9 (C-5, 9); 144.5 (C-3); 157.8 (C-7); 167.8 (C-1).

### 2.3. Emulsion Preparation

The 4:6 (*v*/*v*) corn oil-in-water emulsions were prepared by mixing 4 mL of stripped corn oil containing 1.5 mM of the coumarate ester and 6 mL of an aqueous acid solution (0.04M citrate buffer, pH 3.65) and a weighed amount of non-ionic surfactant Tween 20. The surfactant volume fraction, Φ_I_ = V_surf_/V_emulsion_, was varied from Φ_I_ = 0.005 to Φ_I_ = 0.04. The mixture was stirred with a high-speed rotor (Polytron PT 1600 E, Malters, Switzerland) for 1 min. The freshly prepared emulsions were transferred to a thermostated cell with continuously stirring. The stability of the emulsions was checked visually; no phase separation was observed within 3–4 h, a much longer time than that required to complete the chemical reaction between 16-ArN_2_^+^ and the AOs (see below).

### 2.4. Determination of k_obs_ Values in Intact Emulsions: Derivatization Method

**Scheme 5:**
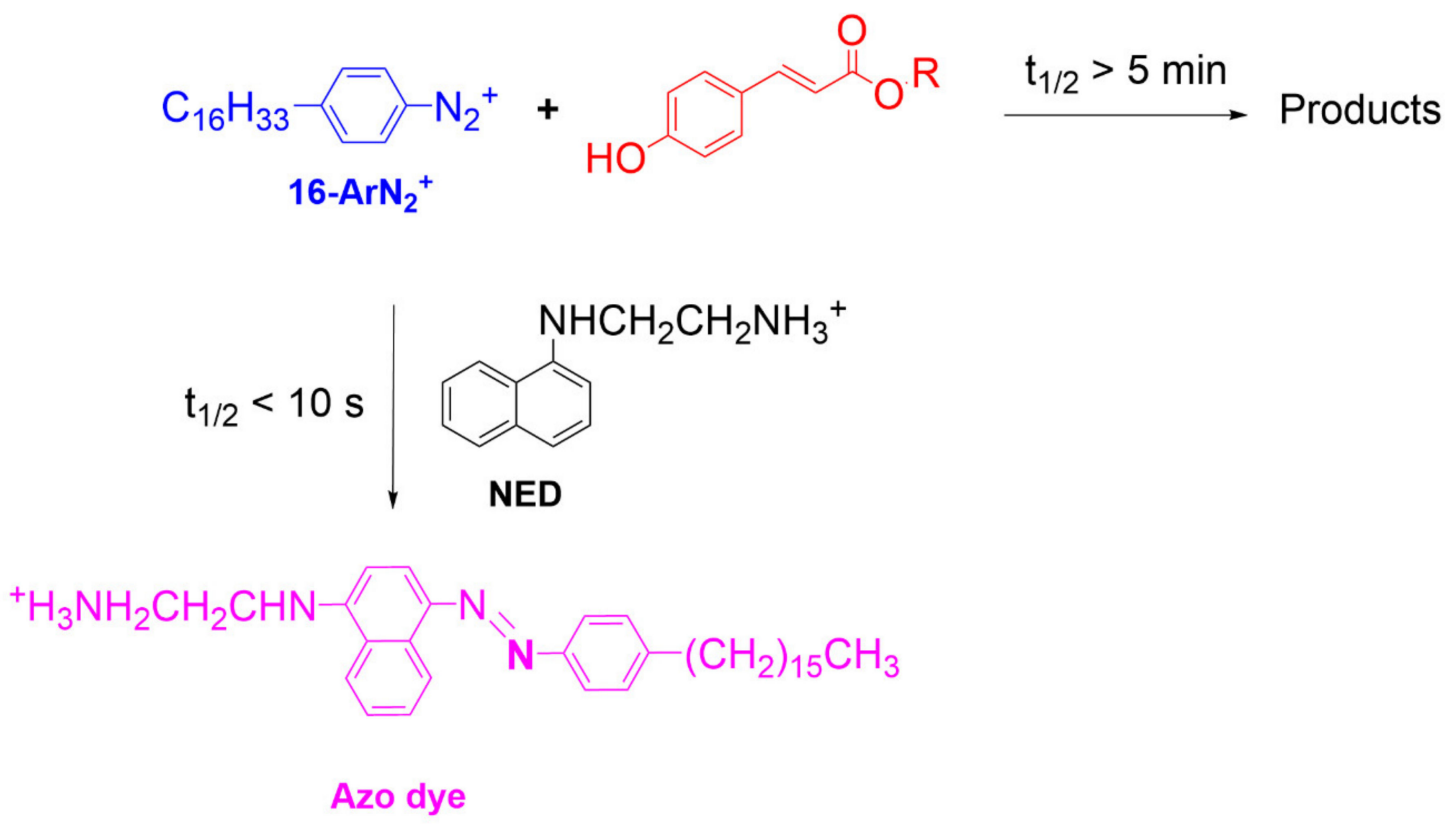
Competitive reactions of 16-ArN_2_^+^ with AOs and with the coupling reagent NED to yield a stable azo dye. Experimental conditions were optimized so that the reaction with NED was much faster than that with AOs, allowing monitoring of the disappearance of 16-ArN_2_^+^ with time in emulsions. Details of the method can be found elsewhere [19,20,38].

The reaction between 16-ArN_2_^+^ and the antioxidants was carried out under pseudo-first order conditions, [16-ArN_2_^+^] <<< [AO]. Emulsions are opaque, and to monitor the disappearance of 16-ArN_2_^+^, a derivatization method that exploits the rapid reaction between the coupling agent *N*-(1-naphthyl)ethylenediamine, NED, and the chemical probe, 4-hexadecylbenzenediazonium, 16-ArN_2_^+^ (Scheme 5) was employed.

**Figure 1:**
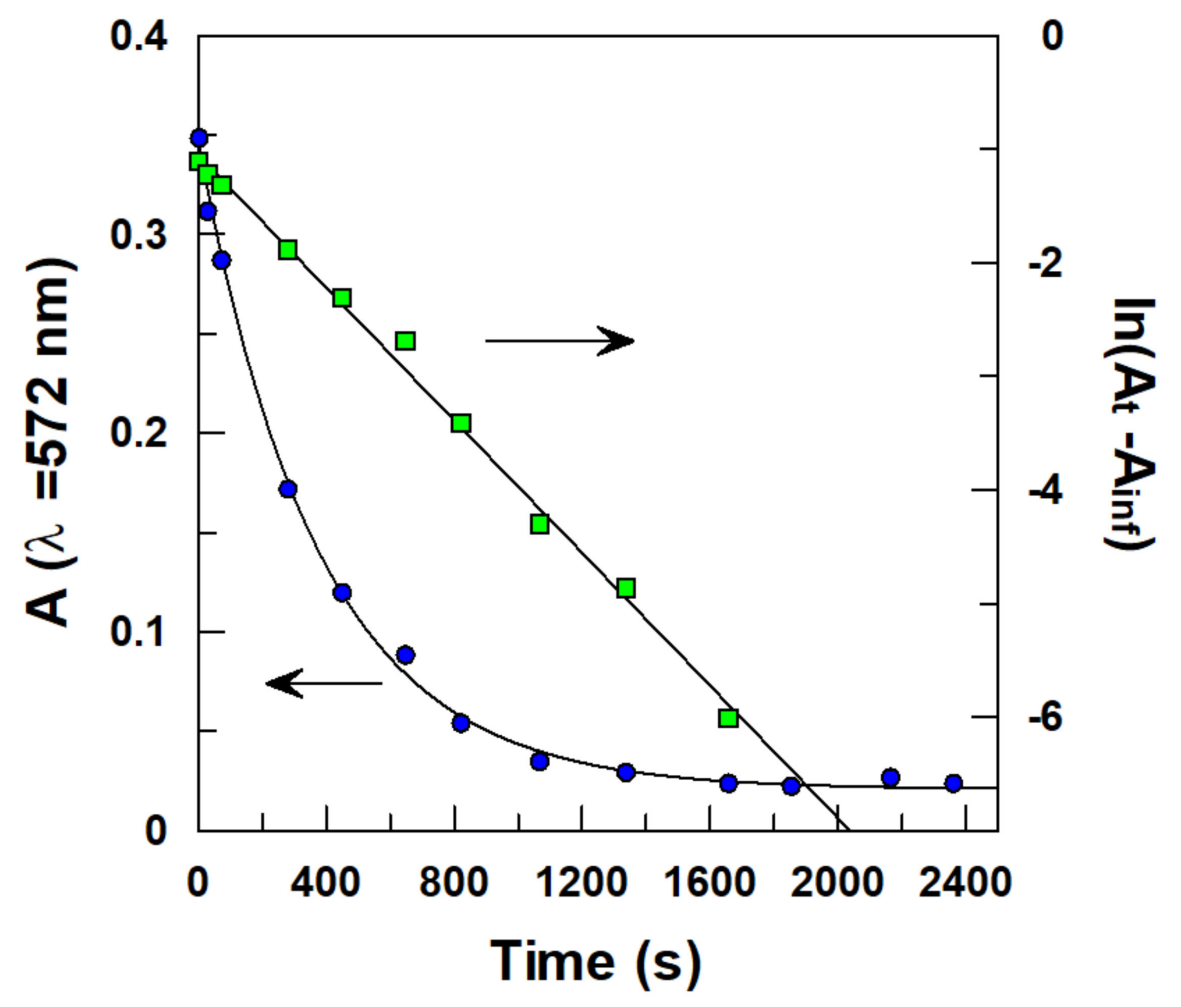
Representative kinetic obtained in corn oil emulsions showing the first-order decrease in the absorbance (λ = 572 nm) of the formed azo dye with time (Scheme 5). The linear variations of ln(A_t_-A_inf_) with time were used to determine *k*_obs_ values for the reaction between 16-ArN_2_^+^ and the antioxidants. The solid lines are the theoretical curves obtained by fitting the (A, t) and (ln(A_t_-A_inf_), t) pairs of data to the integrated and linearized first-order equations. Runs were carried out in the intact emulsions in triplicate, and the average values (deviations less than 6%) were used in calculations. Experimental conditions: 4:6 stripped corn oil/Tween 20/acidic water (citrate buffer 0.04M, pH 3.65) emulsions, Φ_I_ = 0.0084, [16-ArN_2_^+^] = 1.0 × 10^−4^M, [TC] = 2.0 × 10^−3^ M, T = 25 °C.

Experimental conditions were optimized so that the reaction with NED was much faster (half-life t_1/2_< 10 s) than that with the antioxidant (t_1/2_ > 300 s). Briefly, a freshly prepared emulsion (10 mL) was transferred to a thermostated cell. Once the emulsion was thermostated, the reaction between the antioxidant and the probe molecule was initiated by adding an aliquot (16 μL) of a 0.17M stock 16-ArN_2_^+^ solution in acetonitrile under pseudo-first order conditions ([AO] >>> [16-ArN_2_^+^]). Independently, 15 numbered and stoppered test tubes were placed in a thermostatic bath (T = 25 °C), and 2.5 mL of a 0.02 M EtOH-BuOH (50:50, *v*:*v*) solution of NED was added to each test tube and allowed to reach thermal equilibrium. Aliquots (200 µL) of the reaction mixtures were removed at specific time intervals and added immediately to test tubes to initiate azo dye formation. Auxiliary experiments showed that the absorbance of the formed azo dye could be linearly correlated with [16-ArN_2_^+^], and therefore the variation of the absorbance of the azo dye with time could be used to determine indirectly *k*_obs_ by fitting the data to the integrated first order equation, as illustrated in Figure 1. Duplicate or triplicate experiments gave *k*_obs_ values with deviations lower than 7%. Further experimental details as well as advantages and limitations of the method can be found elsewhere [19,37].

### 2.5. Oxidative Stability of Emulsions: Schaal Method

Emulsions were allowed to spontaneously oxidize at T = 55 °C in the dark, and the progress of the oxidation reaction was assessed as in previous works [13,14,15,16,17,18] by monitoring the formation of primary oxidation products with time according to the AOCS Official Method Ti 1a 64. Aliquots (50 µL) of the emulsion were removed at selected times and diluted to 10 mL with ethanol, and the absorbance was determined at λ = 233 nm. Emulsions with no added antioxidant were used as the control, and the relative efficiency of antioxidants was assessed by comparing the time needed to achieve an increase in the formation of conjugated dienes of 0.5%. Experiments were carried out in triplicate, and only the average values are reported.

## 3. Results and Discussion

### 3.1. Oxidative Stability of Corn Oil Emulsions: Effects of Surfactant Concentration

**Scheme 6:**
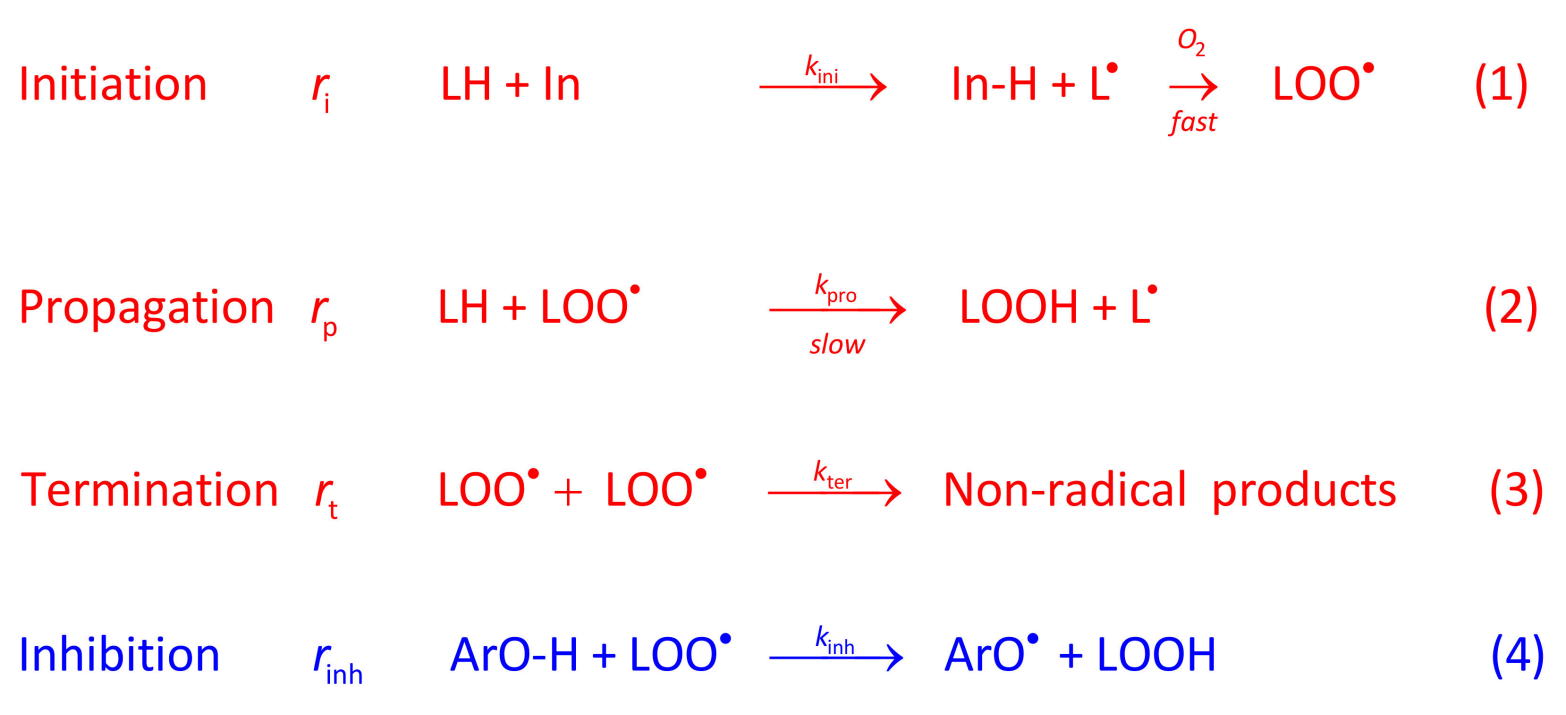
Simplified mechanism for the lipid oxidation reaction comprising the initiation (i), propagation (p), and termination (t) steps. For the sake of simplicity, only the slow (rate-determining) step of the propagation sequence is shown. The oxidation reaction may be hindered by the addition of antioxidants (ArO-H) that regenerate the parent lipid by donation of an H-atom to the peroxyl radical. Further details on the mechanism of the reactions can be found elsewhere [12,13,46]. In: any initiator, LH: unsaturated fatty acid, ArOH: antioxidant, LOO^•^: peroxyl radical, ArO^•^: radical derived from the antioxidant.

**Figure 2:**
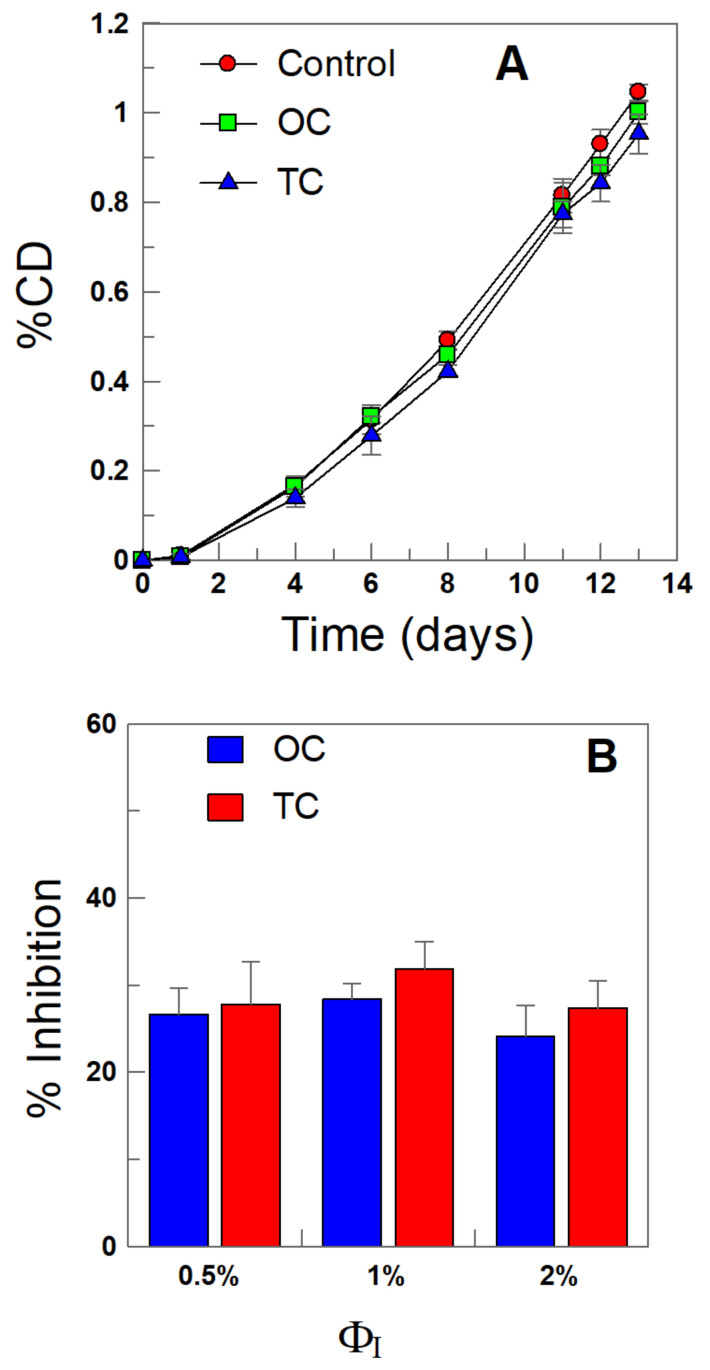
(**A**) Kinetics of production of primary oxidation products in 4:6 corn oil emulsions in the presence and absence of OC and TC (Φ_I_ = 0.01) as determined by the variation in the formation of conjugated dienes with the time. T = 55 °C. (**B**) Percentage of inhibition of OC and TC on the formation of conjugated dienes at different surfactant volume fractions (Φ_I_ = 0.005, 0.01, and 0.02). Values determined by employing Equation (9) with data extracted from Figure 2A (day 13).

To analyze the effects of surfactant concentration on the oxidative stability of corn oil-in-water emulsions, three emulsions with surfactant volume fractions of Φ_I_ = 0.005, 0.01, and 0.02 were prepared, and the formation of primary oxidation products (conjugated dienes, CDs) was monitored with time at T = 55 °C in the presence and absence (control experiments) of AOs; Figure 2A. The kinetic profiles are characterized by a relatively slow buildup of CDs in time followed by a much faster production of CDs (which corresponds to the propagation reaction). A very simplified mechanism of the lipid oxidation reaction is shown in Scheme 6 (reactions 1–3), showing the initiation, propagation, and termination steps.

The reaction is inhibited in the presence of efficient antioxidants because the antioxidant donates an H-atom to the lipid peroxide radicals (reaction 4), a reaction that is competitive with reaction 2. When the antioxidant concentration is nearly depleted, the inhibition reaction becomes uninhibited, and the rate of the overall oxidation reaction increases [5,41,42,43]. On the basis of Scheme 6, one can define efficient antioxidants as those whose rate of trapping radicals, *r*_inh_ (reaction 4) is equal to, or higher than, the rate of radical production *r*_p_, reaction 2 [18,44,45]. The higher *r*_inh_ is, the higher the efficiency is.

Figure 2A shows a typical kinetic plot showing the formation of primary oxidation products (conjugated dienes) with time. The relative efficiency of antioxidants can be assessed by employing Equation (9), which provides the percentage of inhibition of the particular antioxidant compared to the sample in their absence (control sample), and where C is the increment in the percentage of conjugated dienes (%CD) of the control and S is the increment in the %CD of corn oil emulsions in the presence of antioxidant ([AO] = 0.6mM in the oil region) [14].

(9) $$\%\mathrm{Inhibition}\;=\;100\frac{(C-S)}{C}$$

The variation of % Inhibition with the surfactant volume fraction is shown in Figure 2B. As illustrated, both antioxidants have a similar but modest efficiency in inhibiting the lipid peroxidation reaction.

The modest effect of the antioxidants in inhibiting lipid peroxidation can be due to a low value of the rate constant *k*_inh_ or because the concentration of the antioxidants at the reaction site is very low [13,14,15,16,17,18]. It is worth noting that the rate of the inhibition reaction (reaction 4 in Scheme 6) depends on both the inhibition rate constant and the effective concentrations of the antioxidants at the reaction site. In previous works [13,14,15,16,17,44,45,47,48] we demonstrated that i) The main reaction site between antioxidants and lipid peroxyl radicals is the interfacial region of the emulsions, and ii) the oxidative stability of antioxidants correlates with the effective concentrations of the antioxidants in the interfacial region of the emulsion.

To determine which factor (rate constant or effective concentration) is predominant in controlling the rate of the inhibition reaction, we determined the effective concentrations of the AOs in the corn oil emulsions and analyzed the effects of the surfactant volume fraction.

### 3.2. Distribution of OC and TC in Corn Oil Emulsions: Partition Constants

**Figure 3:**
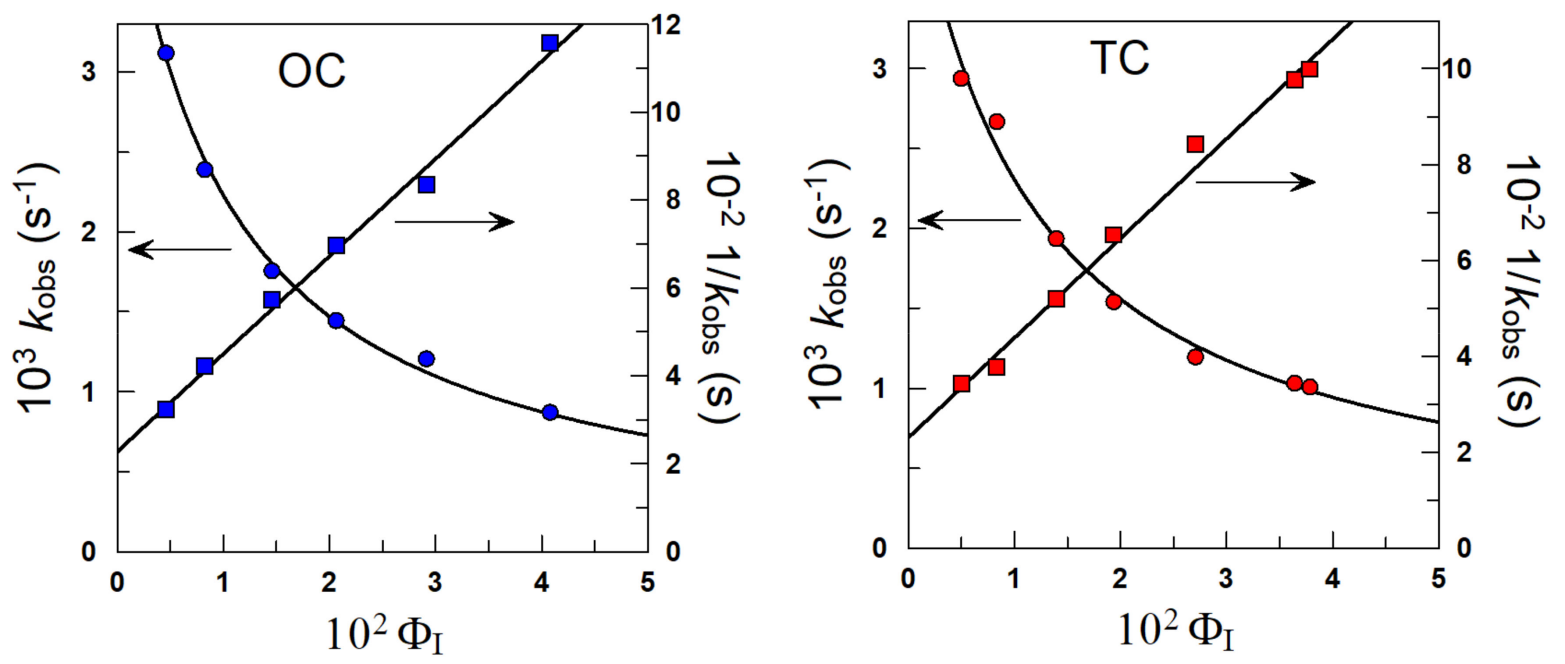
Variation of the observed rate constant *k*_obs_ for the reaction of 16-ArN_2_^+^ with OC and TC in intact 4:6 stripped corn oil-in-water emulsions. Experimental conditions: [16-ArN_2_^+^] = 1.03 × 10^−4^ M, [OC_T_] = 1.54 × 10^−3^ M, [TC_T_] = 9.61 × 10^−4^M, pH = 3.65 (citric/citrate buffer 0.04M), T = 25 °C. The solid lines are the theoretical values obtained by fitting the experimental (*k*_obs_, Φ_I_) and (1/*k*_obs_, Φ_I_) pairs of data to Equations (3) and (4), respectively.

The partition constants of the antioxidants were determined from the variations of the observed rate constant *k*_obs_ with Φ_I_, as described in Section 1.1. Figure 3 shows the variation of *k*_obs_ and 1/*k*_obs_ with Φ_I_ for the reaction between OC and TC with 16-ArN_2_^+^ in 4:6 corn oil-in-water emulsions. Values of *k*_obs_ decrease by a factor of 3–4 from Φ_I_ = 0.005 up to Φ_I_ = 0.04. Such a decrease is in keeping with the predictions of Equation (3). The variations of 1/*k*_obs_ with Φ_I_ are linear, with positive intercepts as shown in Figure 3, in keeping with the predictions of Equation (4).

Values for the partition constants *P*_O_^I^ and for the rate constant in the interfacial region *k*_I_ were determined by employing Equation (4) and are displayed in Table 3. For the sake of comparison, *P*_O_^I^ and *k*_I_ values for the structurally similar caffeic acid derivatives are also given.

*P*_o_^I^ values for OC and TC are higher than one, making the Gibbs free energy for the transfer of the antioxidant from the oil to the interfacial region negative, i.e., ΔG^0,O^^→I^ < 0. Therefore, the investigated antioxidants have a natural tendency to be incorporated into the interfacial region of the emulsion. However, TC has a slightly “lower tendency” than OC (*P*_o_^I^ (TC) < *P*_o_^I^ (OC)) (Table 3) because of its longer alkyl chain, which makes it solubilized preferentially in the oil region rather than in the interfacial region [17,18,49].

Substituents in the aromatic ring of (poly)phenols have a central role in the hydrogen atom-donating ability of phenols, and it may be worthwhile to compare the *P*_o_^I^ values for OC and TC with those for the structurally similar caffeic acid derivatives, where the main difference is the presence of a second –OH group in the aromatic ring in the *m*-position. Only those phenols bearing electron-donating substituents, particularly at the *ortho* and/or *para* positions of the –OH group, are active as antioxidants, because those substituents are expected to lower the phenolic O−H bond dissociation enthalpy and increase the reaction rates with peroxyl radicals. However, addition of a second –OH group not only affects the reactivity of the antioxidant against lipid radicals but also to their partitioning, making them much more hydrophilic. This effect is reflected in the much higher *P*_o_^I^ values obtained for caffeic acid derivatives than for the p-coumaric derivatives. Indeed, the increase in the *P*_o_^I^ values is related to the increase in the intermolecular forces between the antioxidant and the solvent, which are much stronger in the interfacial region than in the oil region because of the presence of water.

Table 3 also shows the values for the rate constant in the interfacial region, *k*_I_, between 16-ArN_2_^+^ and the antioxidants; see Scheme 3. The *k*_I_ values for OC and TC are low and independent of the alkyl chain length, in keeping with results obtained for other antioxidants. Even though *k*_I_ values are not strictly needed to assess the distribution of AOs, their values are important because changes in *k*_I_ values may denote changes in the reaction mechanism. For example, in the present case, the much lower values of *k*_I_ for OC and TC compared to those with octyl and tetradecyl caffeates (ca. 10 fold) are a consequence of the different reaction mechanisms. Reactions with phenols usually proceed through a C-coupling mechanism; meanwhile, in the presence of catechols, arenediazonium ions are reduced through the formation of a diazo-ether intermediate. Relevant mechanistic information can also be obtained from the variations of *k*_I_ with temperature, which provides the activation parameters for the reaction between antioxidants and 16-ArN_2_^+^ and for the changes in *k*_I_ values with acidity, which allow determining whether the reactive species is the anionic, dianionic, or neutral form of the antioxidants.

### 3.3. Distribution of OC and TC between the Oil and Interfacial Regions of the Corn Oil Emulsions

**Figure 4:**
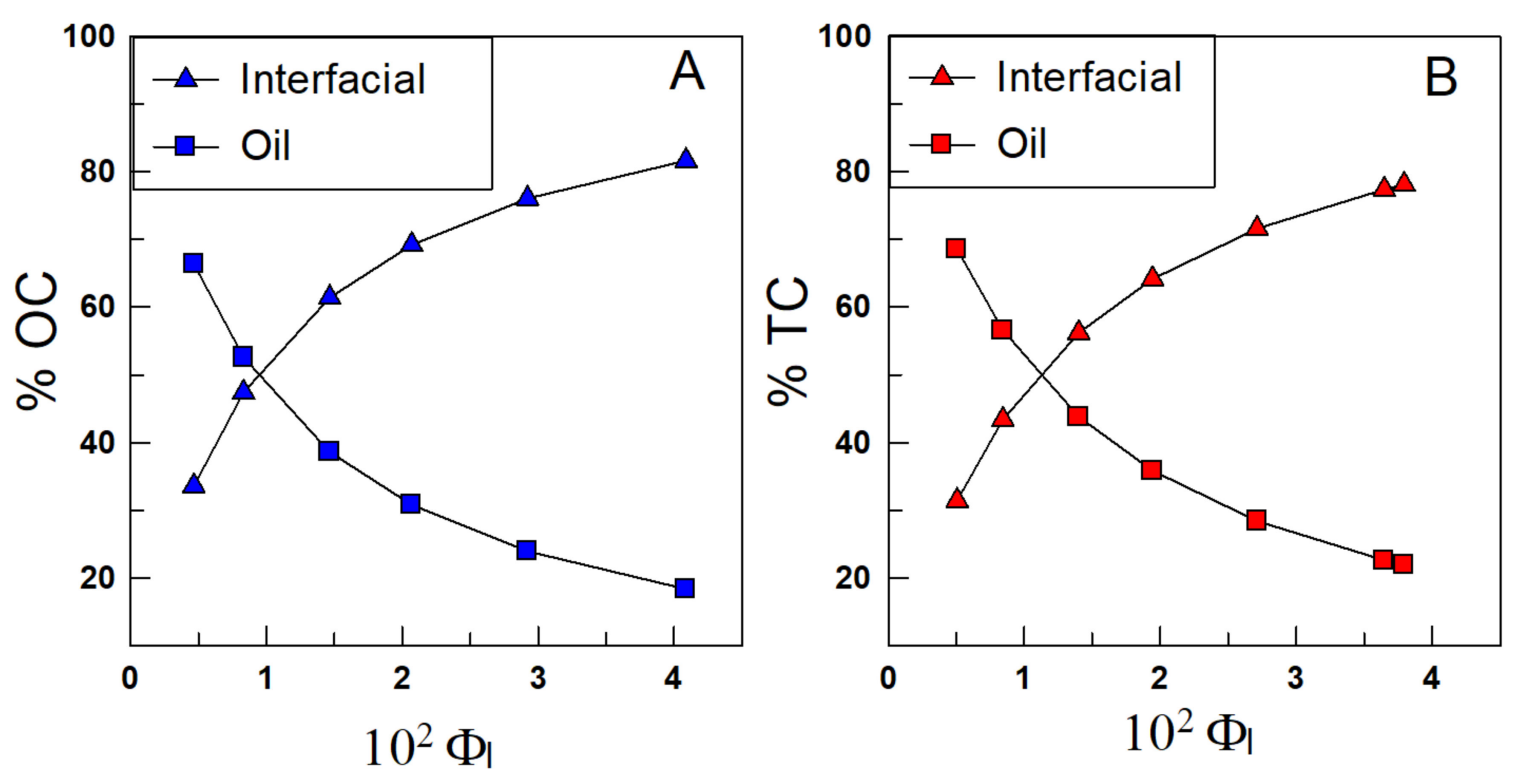
Variation in the percentages of OC (**A**) and TC (**B**) with Φ_I_ obtained in intact 4:6 corn oil-in-water emulsions at T = 25 °C.

The distribution of OC and TC between the oil and interfacial regions of the corn oil emulsions (Scheme 3) was determined by employing the *P*_O_^I^ values in Table 1 and Equations (5) and (6). The variation in the percentage of OC and TC with the surfactant volume fraction Φ_I_ is shown in Figure 4. Results show that at the lowest surfactant volume fraction employed, most of the antioxidants are located in the oil region, about 70%, but their percentage decreases upon increasing Φ_I_, with a concomitant increase in the fraction of AOs in the interfacial region, which goes from ~30% at Φ_I_ = 0.005 to ~80% when Φ_I_ = 0.04. These variations are in line with those determined for other antioxidants, reflecting the tendency of these antioxidants to be preferentially located in the interfacial regions of emulsions.

### 3.4. Effects of Surfactant Concentration and Oil to Water Ratio on the Effective Concentrations of OC and TC in the Oil and Interfacial Regions of Corn Oil Emulsions

**Figure 5:**
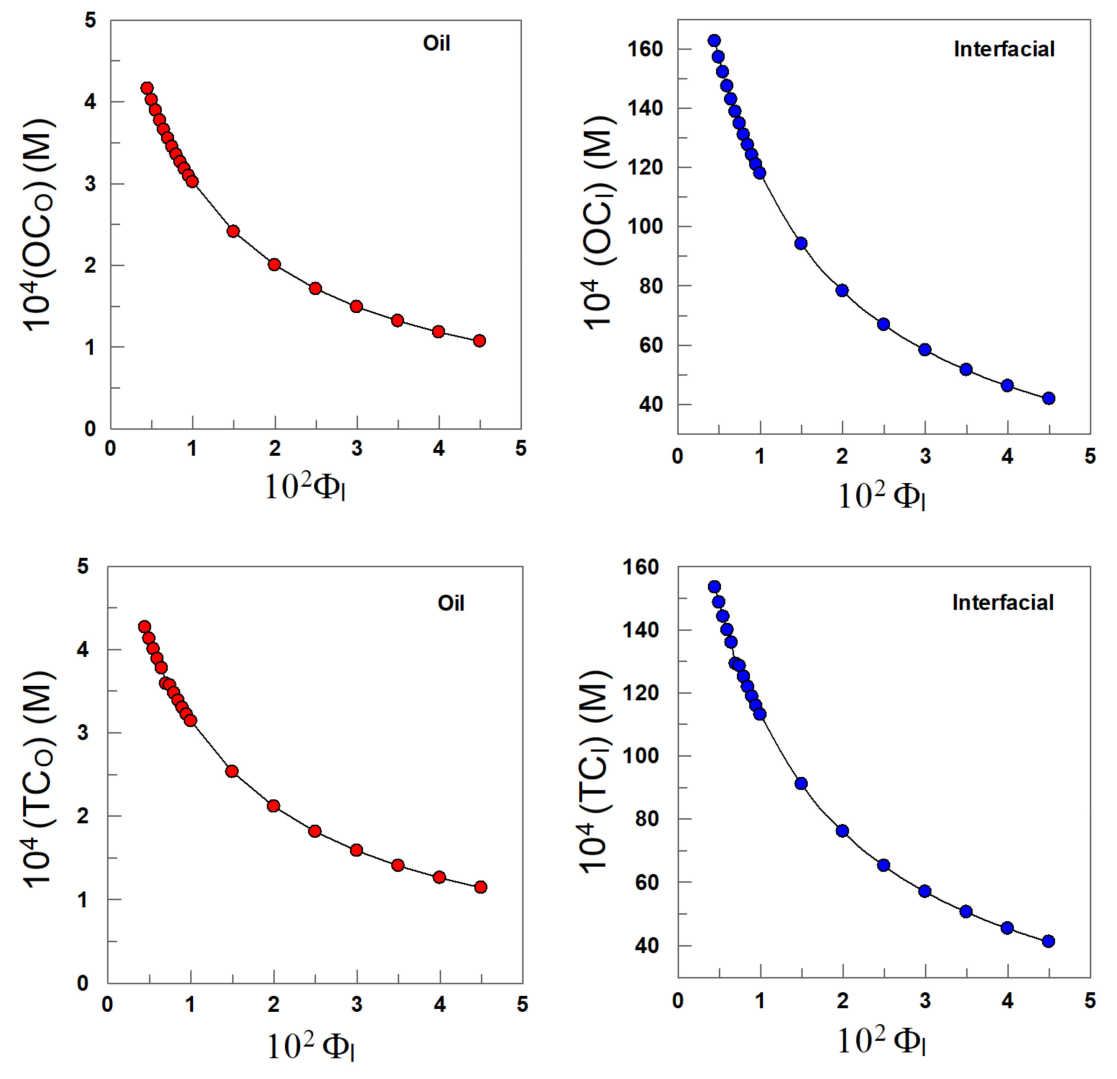
Variations of the effective concentrations of OC and TC in the oil and interfacial regions of corn emulsions with Φ_I_.

Once the distribution of the antioxidants is known, the effective concentrations of the antioxidants in the oil and interfacial regions can be easily determined from the ratio of the moles of antioxidants in each region and the volume of the region; see Equations (7) and (8). Figure 5 shows their variations with the surfactant volume fraction.

The effective molarities of the antioxidants in the interfacial region (expressed in moles per liter of volume of region) are much higher, 2–80 fold, than the stoichiometric concentrations ([AO] = 2 × 10^−4^ M, expressed in moles per liter of emulsion), as a consequence of the accumulation of antioxidants in the region.

At a first glance, the observed decrease in the effective concentrations of OC and TC in the interfacial region upon increasing Φ_I_ may be surprising, because Figure 4 shows that the percentage of both OC and TC in the interfacial region increase upon increasing Φ_I_. This apparent contradiction can be easily explained on the basis of Equations (7) and (8). For one side, %AO_I_ increases upon increasing Φ_I_ from ~30% to ~ 80% —Φ_I_ = 0.005 to Φ_I_ = 0.04—i.e., %AO increases ~2.5 fold. However, upon increasing Φ_I_, the interfacial volume increases eight-fold, diluting the antioxidants. The net result is, therefore, a net dilution of the antioxidants in the interfacial region upon increasing Φ_I_, as observed in Figure 5. The results, however, indicate that both antioxidants are concentrated in the interfacial region at any Φ_I_.

**Scheme 7:**
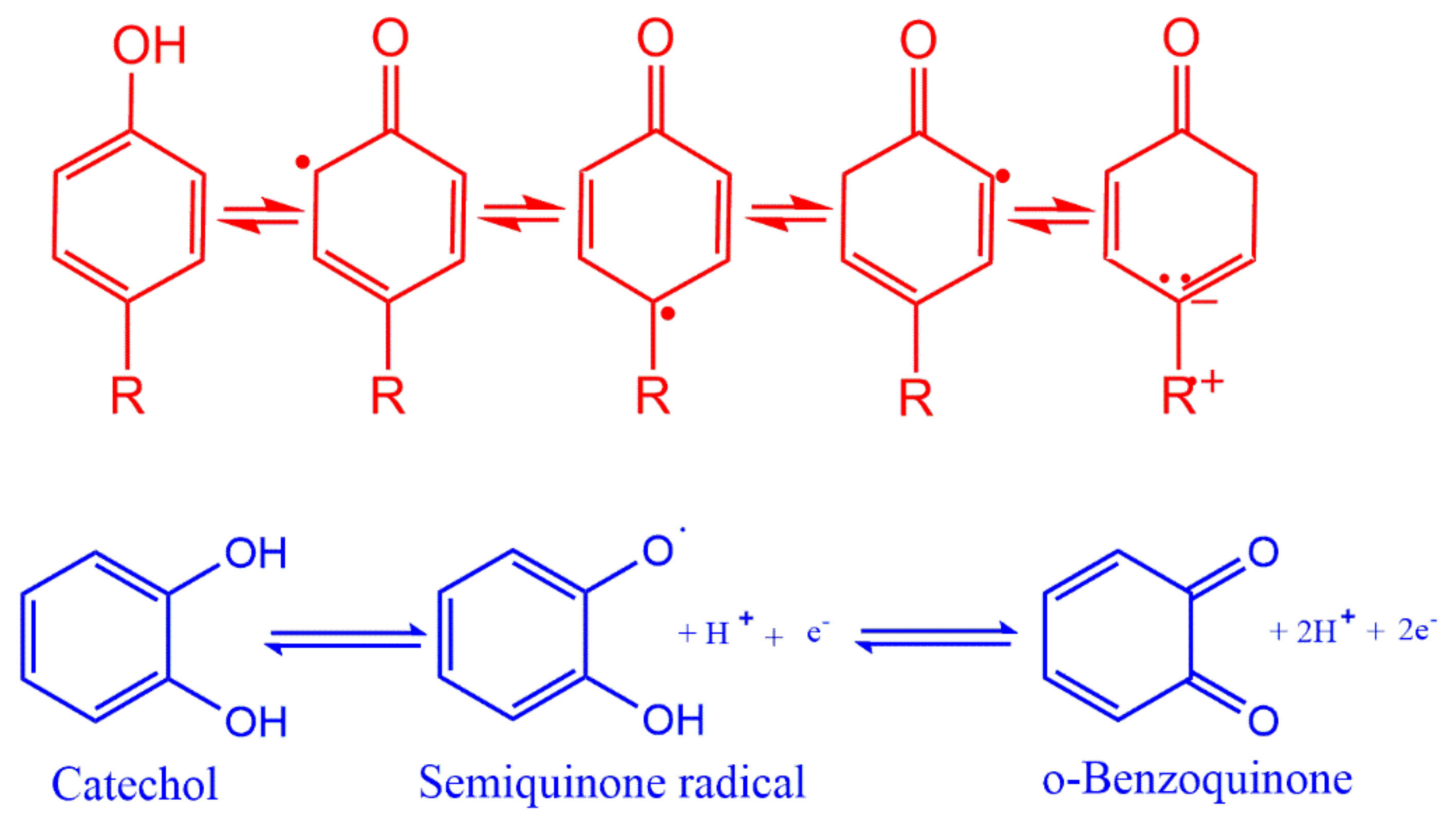
Resonance structures showing the electron delocation in phenol and catechol radicals. R = electron donating group.

Polyphenols react with free radicals through a combination of H-atom transfer (HAT) or electron transfer (SET) mechanisms, and knowledge on the conformational, electronic, and geometrical features of phenolic systems is of crucial importance to understand the relationship between the molecular structure and the antioxidant reactivity. *Ortho* and *para* alkoxyls at the 2, 4, and 6 positions stabilize the phenoxyl radical by inductive and hyperconjugative effects. The conjugative effects of heteroatoms at the *para*-positions provide stabilization through resonance, as shown in Scheme 7. In addition, *ortho* groups may cause some steric hindrance to minimize their effects as pro-oxidants. Catechols, 1,2-dihydroxybenzene, and derivatives are remarkably active antioxidants compared to most *ortho*-methoxyphenols. Overall, an *ortho*-methoxyphenol is somewhat deactivated as an antioxidant by intramolecular hydrogen bonding, whereas catechol derivatives are activated.

On the basis of Scheme 6, one can define as “efficient antioxidants” those molecules whose rate of trapping radicals, *r*_inh_ = *k*_inh_(LOO^•^)(ArOH), is equal to or higher than the rate of radical production *r*_p_ = *k*_p_(LOO^•^)(LH) [18,44,45]. This means that i) the *most reactive antioxidant may not be the most efficient antioxidant* because its effective concentration at the reaction site may not be very high, and ii) the rate of the inhibition reaction *r*_inh_ can be modulated by either modifying *k*_inh_ and/or optimizing its effective concentration in the interfacial region [16,17,18,19]. Our results show, therefore, that the low efficiency of OC and TC in inhibiting the oxidation of the corn oil fatty acids is mainly a consequence of the low reactivity of monophenols towards the lipid radicals and is not due to a decrease in the effective concentration. The results are consistent with published kinetic data showing that the rate constant for the inhibition reaction of 4-methyephenol (*k*_inh_ = 0.97 × 10^−4^ M^−1^ s^−1^) with styrene radicals is ~55 times lower than that for catechol (*k*_inh_ = 55 × 10^−4^ M^−1^ s^−1^) [46,50].

Proper choice of AOs to minimize the oxidation of lipids in emulsions is puzzling, because their efficiency is governed by both their rates of scavenging free radicals and their effective concentrations at the reaction site, which are controlled by their partitioning in the emulsified system. The basis for understanding the effects of substituents on the antioxidant efficiencies of phenols are mostly grounded on the quantitative kinetic studies of absolute rate constants for hydrogen atom transfer from substituted phenols to peroxyl radicals. Comprehensive reviews in bulk systems have been published, and the interested reader is referred [50,51,52], but key aspects relating the structural features of AOs to their efficiencies in emulsified systems are not well known, in part, because there are not direct relationships between the chemical structure of the AOs and their efficiency. For example, inspection of the effects of the alkyl chain lengths for several series of homologous AOs on their efficiency demonstrate that they are parabolic-like, with a maximum at an intermediate (C_4_–C_12_) chain length [19,27,40,53,54]. Thus, an increase in the length of the alkyl chain of AOs changes both their relative solubility in the oil, water, and interfacial regions of the emulsion and their hydrophilic-lipophilic balance (HLB) [19,49,55] and may also modify the AO oxidation/reduction potentials [56]. This parabolic dependence of AO efficiency upon chain length, known as the “cut-off” effect [53,57,58], was reported for the first time more than a century ago when describing both the chemical and biological activity of a series of homologous AOs, and it has been now commonly observed for series of various antioxidants [57,59,60,61,62,63].

## 4. Conclusions

In summary, our results show that hydrophobic coumaric derivatives are not optimal candidates for use as antioxidants because the rate constants of their reaction with lipid radicals are low. This, however, does not hamper their use as bactericides because their hydrophobicity is optimal to locate them in the interfacial and oil regions of emulsions.

Our results also show that the pseudophase kinetic method provides a powerful tool to separate the contributions of the properties of the reaction site (e.g., polarity, reflected in the *k*_I_ values) and those of concentration (reflected in the partition constants). The partition constant *P*_O_^I^ values for OC and TC are higher than one, which means that the Gibbs free energy for the transfer of the AO from the oil to the interfacial region is negative, showing the natural tendency of antioxidants to be located in the interfacial region of emulsions. In general, partitioning of AOs between the different phases or regions of emulsions depends on both the differences in solvation of the solutes by solvent molecules or emulsifiers and on the capabilities of solutes in terms of intra- and intermolecular hydrogen bonding with solvent. The relative importance of each contribution cannot be easily established, and further work is needed to determine distributions and effective concentrations for a large number of AOs under different experimental conditions.

## Figures and Tables

**Table 1:** Typical food colloids and the surfactants employed in their kinetic stabilization. Adapted from ref. [4,7].

Type	Food	Surfactant Employed
o/w	Milk, Sauces	Phospholipids, Proteins
o/w	Parenteral emulsions	Tweens, Proteins
o/w	Mayonnaise	Lecithin
o/w	Bakery products	Proteins
o/w + air	Creams, Ice creams	Sodium casseinate, Carrageenan
w/o	Butter and Margarine	Lecithin

**Table 2:** Applications and Corresponding HLB Values. Adapted from refs. [2,8].

HLB Range	Application
<3	Surface films
3–6	Water-in-oil emulsions
7–9	Wetting agents
8–15	Oil-in-water emulsions
13–15	Detergents
15–18	Solubilizers

**Table 3:** Values of the partition constants *P*_O_^I^ and of the rate constants in the interfacial region, k_I_, for the ocyl and tetradecyl esters of p-coumaric and caffeic acids determined in corn oil emulsions stabilized with Tween 20. Values for the caffeic acid esters were obtained from ref. [47].

Antioxidant	Structure		*P*_O_^I^	*k*_I_ (M^−1^ s^−1^)
*p*-Coumaric Acid Esters	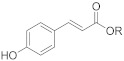	R = C_8_H_17_ (OC)	39.07	0.005
		R = C_14_H_29_ (TC)	36.10	0.005
Caffeic Acid Esters	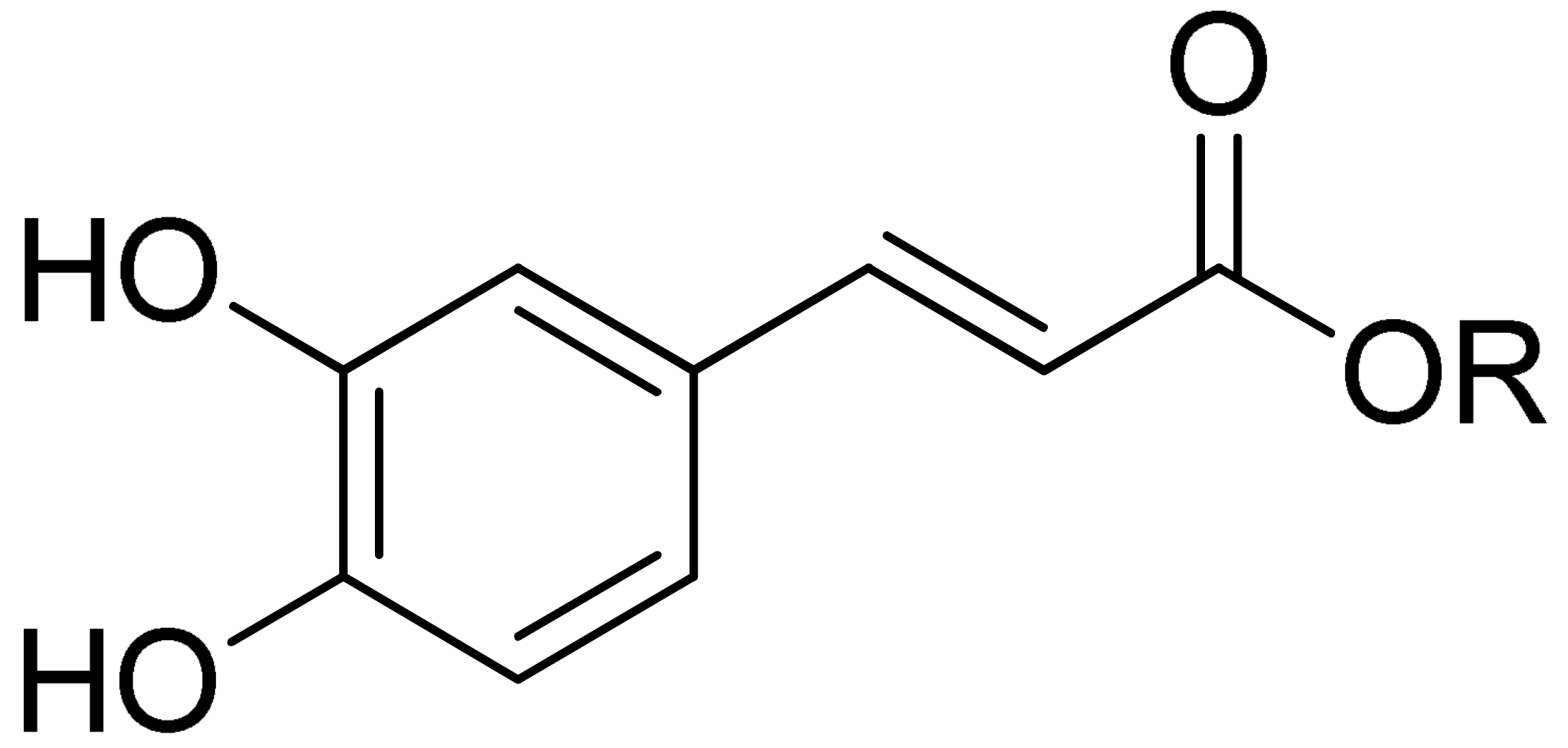	R = C_8_H_17_(C_8_CA)	513	0.053
		R = C_16_H_33_(C_16_CA)	385	0.053

## Data Availability

Not applicable.

## Sample Availability

Samples of the compounds are available from the authors.
